# The influence of different skeletal patterns on TMJ anatomy: a comprehensive CBCT study across various sagittal and vertical skeletal patterns in adults

**DOI:** 10.1007/s00784-026-06916-6

**Published:** 2026-05-22

**Authors:** Sara Eslami, Babak Sayahpour, Ali Tashakor, Elnaz Tavazozadeh, Ari Harsoputranto, Anand Marya, Abdolreza Jamilian

**Affiliations:** 1 Department of Orthodontics, Center for Dentistry and Oral Medicine (Carolinum), Goethe University Frankfurt, Theodor-Stern-Kai 7, 60596 Frankfurt, Germany; 2 Orthodontic Department, Faculty of Dentistry, Tehran Medical Science, Islamic Azad University, Tehran, Iran; 3 Department of Dentistry, School of Dentistry, UCLA, Los Angeles, CA USA; 4 Orthodontic Department, Faculty of Dentistry, University of Puthisastra, Phnom Penh, Cambodia; 5 The City of London Dental School, University of Great Manchester, Bolton, UK; 6 Dentistry (Research) & Program Director of Orthodontics, Faculty of Dentistry, University of Puthisastra, Phnom Penh, Cambodia; 7 Orthodontic Department, Faculty of Dentistry, Tehran Medical Sciences, Islamic Azad University, Tehran, Iran

**Keywords:** Orthodontics, Cone-beam computed tomography, Malocclusion, TMJ anatomy, Skeletal patterns, Skeletal classifications

## Abstract

**Objective:**

This study aimed to evaluate the impact of sagittal and vertical skeletal patterns as well as gender on temporomandibular joint (TMJ) morphology using Cone Beam Computed Tomography (CBCT).

**Methods:**

A cross-sectional analysis was conducted on CBCT scans of 126 adult patients (63 females, 63 males) aged 20 to 40 years, stratified into Class I, II and III sagittal classifications and further divided by vertical patterns: horizontal, normal and vertical. Measurements included condylar dimensions (length, height, and width) and glenoid fossa dimensions (depth and width). Left and right TMJs were assessed and average values were used due to the absence of significant side differences. A multiple linear regression model was used to examine the effects of sagittal and vertical patterns and Gender on the parameter. Gender was included as a covariate in all models. Both an interaction model (vertical × sagittal pattern) and a main-effects-only model were considered with interaction effects accounted for where present. Model selection was based on statistical significance and model fit criteria. Pairwise comparisons were conducted using Tukey’s Honest Significant Difference (HSD) test and estimated marginal means (EMMs) were calculated where applicable. Statistical significance was set at *p* < 0.05 with adjustments for multiple comparisons.

**Results:**

For Condyle Height, a significant difference was found between Class III and Class I within the vertical pattern (VP) subgroup (*p* = 0.011). In contrast, Gender significantly influenced Condyle Length (*p* = 0.009) and a significant difference was found between Class II and Class I (*p* = 0.01). Subgroup analysis revealed a significant difference between Class II and Class I in the VP group (*p* = 0.023). For Condyle Width, a significant interaction between VP and Class III was found (*p* = 0.029). Significant pairwise differences were observed between HP-Class II and VP-Class III (*p* = 0.023), NP-Class II and VP-Class III (*p* = 0.007), and HP-Class III and VP-Class III (*p* = 0.013). Within the NP subgroup, Class II differed significantly from Class I (*p* = 0.044).

Analysis of Glenoid Fossa Width revealed a significant interaction between NP and Class III (*p* = 0.048). Multiple significant pairwise differences were identified, including NP-Class I versus HP-Class II (*p* = 0.002), NP-Class I versus NP-Class II (*p* = 0.004), and VP-Class II versus HP-Class III (*p* = 0.004).

Regarding glenoid fossa depth, a significant interaction between VP and Class III was found (*p* = 0.037), but no pairwise subgroup differences reached statistical significance (all *p* > 0.05).

**Conclusions:**

This study demonstrates that TMJ morphology is variably influenced by vertical and sagittal skeletal patterns and gender. Significant differences in condyle length and width as well as glenoid fossa width were primarily observed in individuals with vertical patterns and Class II or III skeletal relationships. Gender significantly affected condyle length. While glenoid fossa depth showed a high interaction between vertical pattern and sagittal skeletal relationships, no individual comparisons reached statistical significance. These findings underscore the nuanced interplay of sagittal and vertical skeletal patterns in shaping TMJ morphology.

## Introduction

With the increasing integration of Cone Beam Computed Tomography (CBCT) in orthodontic practice, including virtual treatment planning for clear aligners, indirect bonding and combined orthodontic-surgical cases, its diagnostic value has become indispensable [1, 2]. Legal and ethical guidelines emphasize the need for practitioners to diagnose and document all anatomical structures captured within the CBCT field of view, enhancing its utility beyond conventional imaging methods. CBCT is a widely used modality for assessing temporomandibular joint (TMJ) anatomy due to its ability to provide accurate linear and volumetric measurements with minimal superimposition [3]. Unlike traditional two-dimensional methods, CBCT enables accurate visualisation of bone structures with minimal overlap, amplification or distortion, making it particularly suited for diagnosing TMJ pathologies and structural variations [3–6]. 

The TMJ is one of the body’s most anatomically and biomechanically complex joints. Its morphology significantly influences orthodontic and orthognathic treatment outcomes and long-term stability [7–9]. Condylar dimensions and their positional relationships within the glenoid fossa are critical components of comprehensive orthodontic treatment planning. Studies suggest that larger condyles may offer more stable support for occlusal changes and greater resistance to pathological alterations. In contrast, smaller condyles are often associated with instability and increased susceptibility to displacement and dysfunction [6, 10]. 

As more adults pursue orthodontic treatment, often as their second or third intervention following previous orthodontic care during their youth, prioritizing the health and stability of the TMJ is becoming increasingly essential [11]. Many patients presenting for treatment may already exhibit temporomandibular disorders (TMDs), underscoring the importance of incorporating TMJ considerations into treatment planning. Normalizing the condylar position and understanding its morphology during treatment planning can significantly improve outcomes. This necessitates a thorough understanding of normal TMJ variations to avoid misdiagnosis and optimize patient care.

However, evidence regarding the relationship between TMJ morphology and factors such as gender, sagittal skeletal classification and vertical skeletal patterns remains inconsistent [5, 6, 8, 12–23]. While some studies report significant associations between condylar dimensions and these variables, others fail to establish such correlations. Methodological limitations, including heterogeneous samples combining adults with growing patients, unbalanced gender representation and insufficient statistical power, have contributed to these discrepancies.

Given these challenges, the present study aimed to comprehensively evaluate condylar and glenoid fossa dimensions in adults with different sagittal (Class I, II and III) and vertical (horizontal, normal and vertical) skeletal relationships, stratified by gender. This approach helps clarify previous inconsistencies by analyzing interaction effects that are seldom explored in the literature.

## Methods and materials

This cross-sectional study was conducted on Cone Beam Computed Tomography (CBCT) scans retrieved from the archives of the Faculty of Dentistry at Tehran Azad University and a private oral and maxillofacial radiology office. All procedures adhered to the ethical standards set by the committee responsible for human experimentation (institutional and national) and the Helsinki Declaration of 1975, as revized in 2013. The Ethics Commission of Tehran Azad University granted ethical approval for this study under the identifier IR.IAU.DENTAL.REC.1401.115. The archives were retrospectively searched from January 1, 2024, backwards until the required sample size for each classification category (126 patients in total, 42 in each group and 14 in each subgroup) was achieved, reaching as far back as November 5, 2019.

126 CBCT images of patients aged 20 to 40 were analyzed. These images were originally acquired for diagnostic or treatment purposes unrelated to the present study. All scans were obtained using a Sirona Galileos device (Dentsply Sirona, Bensheim, Germany) under the Scan-Fast protocol.Acquisition parameters were as follows: exposure time of 14 s, tube voltage of 98 kV, and tube current of 3 mA, with a field of view (FOV) of 15 × 15 cm encompassing both TMJs. The voxel size was 0.3 mm, with a reconstruction filter optimized for bone visualization and a slice thickness equal to the voxel dimension. Images were reconstructed using a 512 × 512 matrix, and cephalometric projections were generated from the CBCT volume data.

Participants were positioned upright, with the Frankfurt horizontal plane parallel to the floor and the midsagittal plane perpendicular to the detector. Each patient was instructed to maintain the head posture by looking into a mirror during scanning, with teeth in maximum intercuspation. Image datasets were exported and analyzed using Dolphin Imaging software, version 11.9 (Dolphin Imaging & Management Solutions, Chatsworth, CA, USA).

### Participant selection

Inclusion criteria included systemically healthy adults aged 20 to 40 with different skeletal patterns in sagittal (skeletal class I, II and III relationship) and vertical (horizontal, moderate and vertical skeletal pattern) dimensions. Exclusion criteria encompassed a history of temporomandibular disorders (TMDs), TMD treatment including TMJ surgery or splint therapy, presence of osteophytes or condylar flattening, previous functional, orthodontic or orthosurgical treatments, systemic diseases, inflammatory joint disease, facial or mandibular asymmetry, history of trauma, facial fractures, or craniofacial syndromes.

### Sample stratification

The 126 consecutively recruited samples were divided into three sagittal classification (class I, II, and III) groups, each containing 42 samples. They were further stratified into vertical, moderate, and horizontal skeletal subgroups, with 14 samples per subgroup. Each subgroup was subsequently divided by gender, with 7 male and 7 female samples per subgroup.

Sagittal skeletal classifications were determined using the ANB angle and Wits value [4]. :

1. Class I: 0° ≤ ANB ≤ 4°, -1 ≤ Wits ≤ 0.
2. Class II: 4° < ANB, 0 < Wits.
3. Class III: ANB < 0°, Wits < -1.

Vertical skeletal patterns were classified using the SN-GoGn angle [18]. :

1. Horizontal pattern (HP): SN-GoGn < 27°.
2. Normal pattern (NP): 27° ≤ SN-GoGn ≤ 37°.
3. Vertical pattern (VP): 37° < SN-GoGn.

### Measurement protocol

All CBCT scans were converted to DICOM format and analyzed using Dolphin Imaging software (Management & Imaging Solutions, Chatsworth, CA, USA). Condylar dimensions were measured bilaterally (right and left sides) on each scan using parameters initially described by Hilgers et al. [24] and subsequently adopted by various researchers in similar studies [16, 25]. This method relies on identifying anatomical landmarks and measuring the distances between them.

1. Condylar Dimensions (Fig. 1):
  **Condylar Length**: Measured in the sagittal view as the linear distance between the posterior condylar point (PCo) and anterior condylar point (ACo), 4 mm below the highest condylar point (SCo).
  **Condylar Height**: Calculated as the vertical distance from a tangent drawn at the lowest sigmoid notch point, parallel to the true horizontal line, to SCo.
  **Condylar Width**: Measured in the coronal view as the linear distance between the medial condylar point (MCo) and lateral condylar point (LCo).

**Fig. 1:**
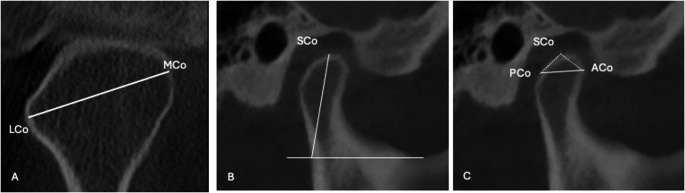
Dimensions of the condyle: (**A**) the width, (**B**) the height and (**C**) the length of the condyle
  The measurement method used by previous studies [22, 26, 27] was used to measure the glenoid fossa dimensions on each side independently.
2. Glenoid fossa dimensions (Fig. 2):
  **Fossa depth**: Measured in the sagittal view from the highest point of the glenoid fossa to the plane formed by the lowest point of the articular eminence and the lowest point of the internal acoustic meatus.
  **Fossa width**: Determined as the linear distance between the lowest points of the articular eminence and the internal acoustic meatus.

**Fig. 2:**
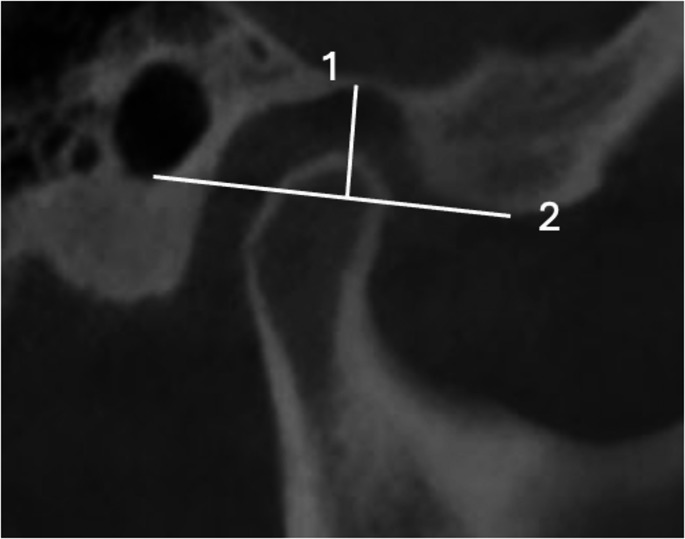
Glenoid fossa dimensions: (1) depth of the glenoid fossa, (2) width of the glenoid fossa

All measurements were performed in a blinded manner. Both examiners were unaware of the patients’ skeletal classification during analysis.

### Statistical analysis

All statistical analyzes were performed using R software (version 4.2.3 ).

The Kolmogorov-Smirnov test confirmed the normal distribution of the data.

The paired t-test was used to analyze data from each group’s left and right TMJs. Since no significant differences were detected between the left and right sides, the average value was used for comparisons.

A multiple linear regression model was used to examine the effects of vertical pattern, sagittal classification and gender on glenoid fossa depth. Gender was included as a covariate in all models. Both an interaction model (vertical pattern × sagittal classification) and a main-effects-only model were considered, with interaction effects accounted for where present. Model selection was based on statistical significance and model fit criteria.

Pairwise comparisons were conducted using Tukey’s Honest Significant Difference (HSD) test, and estimated marginal means (EMMs) were calculated where applicable. Statistical significance was set at *p* < 0.05, with adjustments for multiple comparisons. Data visualization was performed using boxplots to illustrate group differences.

Intra-examiner reliability was calculated using a two-way random effects model (absolute agreement) on 20 randomly selected scans remeasured after a 10-day interval. Intra-examiner ICC was 0.89 (95% CI: 0.82–0.95). Inter-examiner ICC was 0.87 (95% CI: 0.80–0.93).

### Sample size calculation

The statistical power of the present study was calculated based on the results of Chae et al. [15], using the Fixed Effects ANOVA power analysis at α = 0.05. To detect a significant difference in the skeletal pattern variable (horizontal vs. normal vs. vertical) with an effect size of 0.646 at a power exceeding 99%, a sample size of 7 per subgroup was required. For the sagittal skeletal classification variable (class I vs. II vs. III), an effect size of 0.338 required a power of 93%, necessitating seven samples per subgroup. Similarly, to identify differences in the gender variable with an effect size of 1.87 and a power exceeding 99%, the same sample size was sufficient. Consequently, a total sample size of 126 was determined, with 42 samples in each sagittal classification group, 14 samples in each vertical skeletal subgroup and seven samples per gender per subgroup.

## Results

**Fig. 3:**
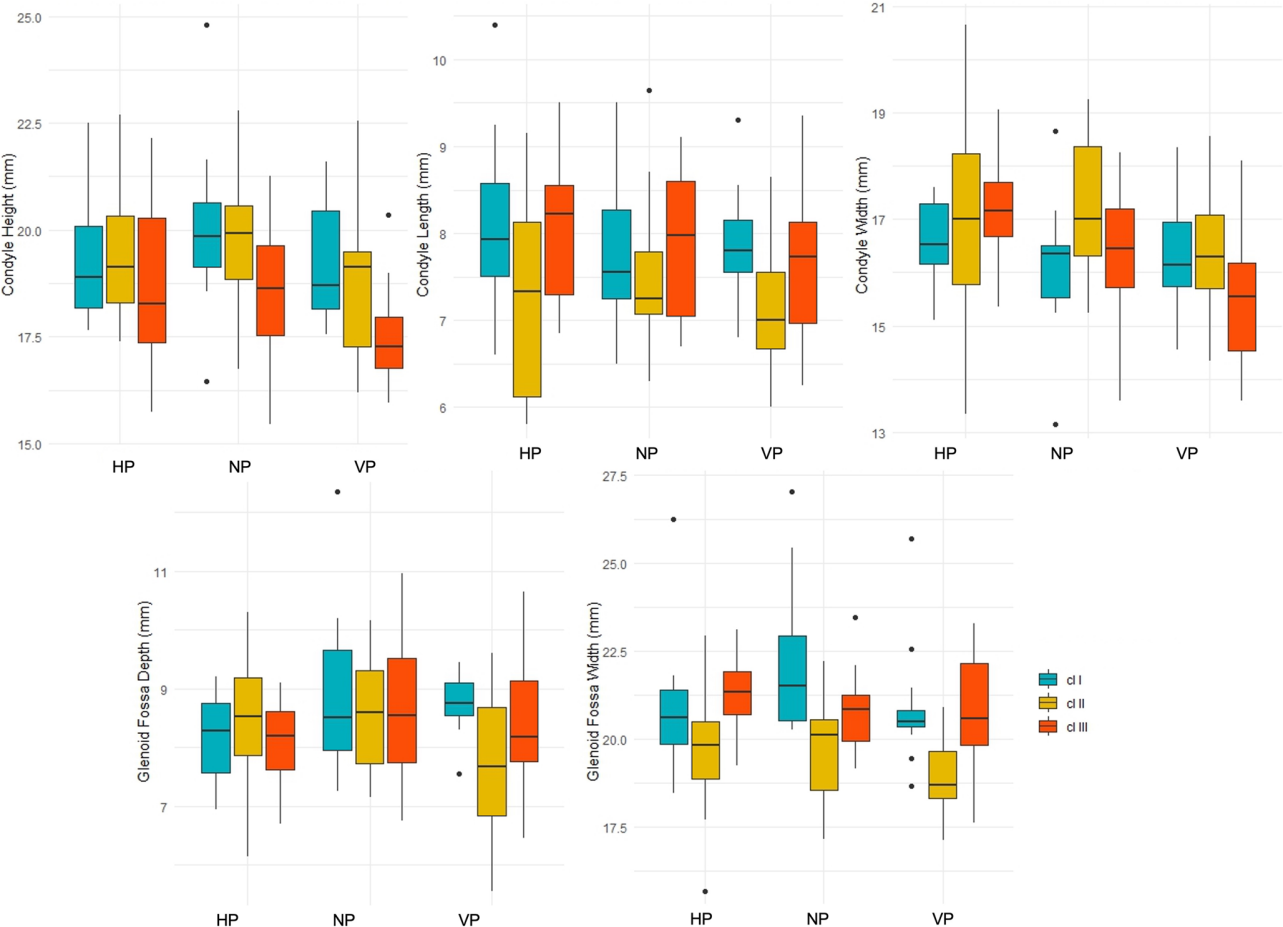
Boxplots showing the distribution of condylar and glenoid fossa dimensions stratified by sagittal skeletal classification (Class I, II, III) and skeletal pattern (HP, NP, VP)

This study included 252 TMJs from 126 participants (63 women and 63 men) with a mean age of 29.90 ± 6.68 years. The participants were evaluated for condylar dimensions (length, width, and height) and the width and depth of the glenoid fossa on both the left and right sides. The data was stratified based on facial skeletal type in the vertical dimension, skeletal classification in the sagittal dimension, and gender. A descriptive analysis of their measurements is shown in Tables 1, 2 and 3; Fig. 3.

**Table 1:** Comparison of the condylar length, height and width as well as depth and width of glenoid fossa in Class I, II and III samples

Variable	Sagittal skeletal relationship	Mean (SD)	*P* value
Condylar length	Class I	7.91 (0.9)	*P* = 0.002
	Class II*	7.28 (0.93)*	
	Class III	7.84 (0.85)	
Condylar width	Class I	16.37 (1.03)	*P* = 0.073
	Class II	16.34 (1.45)	
	Class III	16.92 (1.36)	
Condylar height	Class I*	19.55 (1.60)*	*P* = 0.01
	Class II	19.33 (1.71)	
	Class III	18.88 (1.63)	
Glenoid fossa width	Class I	21.33 (1.92)	*P* < 0.001
	Class II*	19.44 (1.45)*	
	Class III	20.99 (1.40)	
Glenoid fossa depth	Class I	8.61 (0.99)	*P* = 0.503
	Class II	8.22 (1.16)	
	Class III	8.43 (1.16)	

*Statistical significance is set at *p* < 0.05

*SD* standard deviation

**Table 2:** Comparison of the condylar length, height and width as well as depth and width of glenoid in various vertical skeletal patterns

Variable	Skeletal pattern	Mean (SD)	*P* value
Condylar length	NP	7.70 (0.87)	*P* = 0.451
	HP	7.79 (1.06)	
	VP	7.54 (0.81)	
Condylar width	NP	16.57 (1.27)	*P* = 0.016
	HP	16.93 (1.32)	
	VP	16.12 (1.24)*	
Condylar height	NP	19.43 (1.72)	*P* = 0.065
	HP	19.15 (1.64)	
	VP	18.57 (1.74)	
Glenoid fossa width	NP	20.93 (1.88)	*P* = 0.164
	HP	20.63 (1.71)	
	VP	20.21 (1.76)	
Glenoid fossa depth	NP	8.70 (1.18)	*P* = 0.227
	HP	8.21 (0.89)	
	VP	8.35 (1.19)	

*Statistical significance is set at *p* < 0.05

*SD* standard deviation, *NP* normal skeletal pattern, *HP* horizontal skeletal pattern, *VP* vertical skeletal pattern

**Table 3:** Interaction effect between vertical and sagittal skeletal patterns and dimensions of condyle and glenoid fossa

Variable	Class I			Class II			Class III		*P* value
	NP	HP	VP	HP	VP	NP	HP	VP	
Condylar length	7.74 (0.8)	8.08 (1.0)	7.93 (0.5)	7.23 (1.1)	7.11 (0.7)	7.86 (0.8)	8.07 (0.8)	7.60 (0.8)	*P* = 0.031*
Condylar width	16.09 (1.2)	16.60 (0.8)	16.41 (1.0)	17.06 (1.8)	16.48 (1.1)	16.41 (1.1)	17.14 (1.0)	15.47 (1.3)	*P* = 0.007*
Condylar height	19.88 (1.8)	19.32 (1.4)	19.45 (1.4)	19.36 (1.6)	18.81 (1.9)	18.61 (1.5)	18.77 (1.8)	17.45 (1.2)	*P* = 0.005*
Glenoid fossa width	22.21 (2.1)	20.91 (1.8)	20.88 (1.6)	19.64 (1.7)	18.95 (1.1)	20.83 (1.1)	21.34 (1.1)	20.81 (1.8)	*P* < 0.001*
Glenoid fossa depth	8.79 (1.3)	8.16 (0.7)	8.88 (0.6)	8.36 (1.1)	7.74 (1.2)	8.76 (1.3)	8.10 (0.7)	8.43 (1.3)	*P* = 0.113

*Statistical significance is set at *p* < 0.05

*SD* standard deviation, *NP* normal skeletal pattern, *HP* horizontal skeletal pattern, *VP* vertical skeletal pattern.

The left and right side measurements had no statistically significant differences in all three measurements. Therefore, the average value of the left and right sides was used for the inter- and intragroup comparisons. A previous study also employed this method [28]. 

This study employed linear models to investigate the effects of skeletal vertical pattern, angle sagittal classification, and gender (as a covariate) on various craniofacial measurements, including condyle height, condyle length, condyle Width, glenoid fossa width, and glenoid fossa depth. For condyle height, no significant effects were observed for angle sagittal classification or interaction terms overall, although a significant difference was found between Class III and Class I within the vertical pattern (VP) subgroup (*p* = 0.011), with a marginal effect in the horizontal pattern (HP) subgroup (*p* = 0.071). Gender was not a significant factor in this model.

In contrast, gender significantly influenced condyle length (*p* = 0.009), and a significant difference was found between Class II and Class I (*p* = 0.01), while vertical skeletal pattern and interaction terms were not significant. Subgroup analysis revealed a significant difference between Class II and Class I in the VP group (*p* = 0.023), and a near-significant effect in HP (*p* = 0.072).

For condyle width, a significant interaction between VP and Class III was found (*p* = 0.029). Significant pairwise differences were observed among several group combinations, notably between HP-Class II and VP-Class III (*p* = 0.023), NP-Class II and VP-Class III (*p* = 0.007), and HP-Class III and VP-Class III (*p* = 0.013). Within the normal pattern (NP) subgroup, Class II differed significantly from Class I (*p* = 0.044).

Analysis of Glenoid Fossa Width revealed a significant interaction between NP and Class III (*p* = 0.048). Multiple significant pairwise differences were identified, including NP-Class I versus HP-Class II (*p* = 0.002), NP-Class I versus NP-Class II (*p* = 0.004), and VP-Class II versus HP-Class III (*p* = 0.004). Additional significant contrasts were observed within all pattern subgroups.

Finally, for glenoid fossa depth, a significant interaction between VP and Class III was found (*p* = 0.037), but no pairwise subgroup differences reached statistical significance (all *p* > 0.05).

## Discussion

The present study assessed the effects of sagittal skeletal classification (Class I, II, and III), vertical skeletal pattern type (VP, NP, and HP) and gender (female vs. male) on temporomandibular joint (TMJ) parameters, specifically focusing on condylar length, height, width, and the depth and width of the glenoid fossa.

CBCT images of 126 patients were analyzed, with both TMJs measured for each patient. No statistically significant differences were observed between the left and right side measurements across all parameters. Consequently, the average values of the left and right sides were utilized for inter- and intragroup comparisons. This approach aligns with the methodology employed by Noh et al., who found no significant differences between the sides [28]. Although many researchers have found no significant differences between the right and left sides [16, 21, 29–31], others have reported significant differences between sides [15, 19], fueling debate. The absence of such differences in our study may be attributed to the exclusion of asymmetrical cases.

The relationship between sagittal skeletal classification and TMJ morphology remains controversial. In contrast, some studies report minimal or no effects [15, 20, 32, 33]. Others identify significant correlations [14, 16, 18, 28, 31]. The majority suggested higher condylar width and length in Class III individuals [16, 18, 28]. And lower dimensions in Class II patients [16, 22]. In our study, patients with Class II skeletal classification exhibited significantly smaller condylar length compared to Class I group, especially in those with vertical skeletal pattern.

The width of the glenoid fossa was also significantly smaller in patients with Class II classification compared to those with Class I and Class III groups. Conversely, Class III individuals demonstrated a significantly reduced condylar height in patients with vertical pattern compared to those with Class I classification. Our findings align partially with previous studies, showing significantly smaller condylar length and fossa width in Class II patients.

However, unlike prior studies, we did not observe generally larger condylar dimensions in Class III compared to Class I. Only individuals with Class III classification and horizontal pattern had a significantly larger glenoid fossa width than those of Class II samples with vertical pattern. These differences could be attributed to factors such as racial variations in the study populations and heterogeneity in previous samples due to the inclusion of growing patients [18, 22]. There is also the lack of stratification by vertical skeletal patterns in several studies [22, 31]. 

The lower condylar length and fossa width observed in Class II individuals in our study may indicate a higher risk of temporomandibular dysfunction (TMD) in this group since more prominent condyles are known to provide more stable support and greater resistance to disc dislocations compared to smaller condyles [6, 10]. Furthermore, it has been hypothesized that insufficient fossa dimensions can facilitate articular disc displacement [6]. Nonetheless, this statement should be taken with a grain of salt, as the lack of TMD in our study was established through medical history and not by means of clinical examinations. Therefore, our hypotheses regarding TMD associations are only exploratory.

Unlike sagittal skeletal classification, the influence of vertical skeletal patterns on condylar dimensions has been widely acknowledged by many authors. Most studies report smaller condylar dimensions in patients with vertical skeletal patterns compared to those with normal or horizontal skeletal patterns [6, 12, 16, 18, 28]. Our findings are partially consistent with this trend, as we observed significantly smaller condylar width and height in patients with vertical patterns. Notably, a strong correlation was found between vertical pattern Class III regarding the condylar width as well as neutral pattern and Class III regarding the glenoid fossa width. This finding is rarely analyzed, as such an evaluation has not been conducted in previous research. It suggests a potential additive effect of the skeletal pattern on condyle and glenoid fossa width in Class III patients, warranting further investigation in Class III patients.

Comparison of interactions between sagittal and vertical dimensions showed that within the Class III group, significantly reduced condylar width was observed in patients with vertical pattern in our study sample. Interestingly, the vertical skeletal pattern in Class III patients may act as a masking factor, reducing the sagittal prominence by backward mandibular rotation. However, this same vertical trajectory may correlate with suboptimal TMJ loading. The observed reduction in condylar width among Class III vertical subjects suggests a biomechanical environment that hinders transverse condylar development, possibly indicating a more severe, less adaptive phenotype despite a less conspicuous sagittal profile.

Sexual dimorphism in condylar dimensions has also remained a contentious topic in the literature, with several studies reporting smaller condylar dimensions in females compared to males [16, 17, 19, 29, 34], While others have found no significant differences between genders [15, 18, 21, 35]. Our findings partially align with studies suggesting dimorphism, as female patients in our study exhibited lower condylar length values than males. Interestingly, a higher prevalence of temporomandibular dysfunction (TMD) has been reported in female patients, as well as in those with Class II malocclusions and vertical skeletal patterns [5, 6], Overlapping with the groups in our study that exhibited significantly smaller condylar length. This suggests that reduced condylar length could potentially be a predisposing risk factor for TMD in these populations.

## Limitations

The retrospective design certainly introduces some level of selection bias despite blindness of the examiners. Lack of clinical patient examination is another important limitation, as diagnosis of TMD was established merely based on patients’ medical history. Although factors such as bruxism, asymmetry and TMD were exclusion criteria, the absence of formal clinical or radiographic TMD assessment may have allowed undetected cases. Additionally, voxel size and head positioning variations were not standardized due to data availability, potentially affecting measurement precision. Future prospective studies accompanied with clinical examination would further clarify the association between TMD, condylar dimensions and skeletal classifications.

## Conclusions

Following conclusions can be drawn based on the results of the present study:

1. Individuals with Class II skeletal relationships exhibited significantly smaller condylar length and glenoid fossa width than those with Class I and III, especially in those with vertical pattern.
2. Within the Class III group, significantly reduced condylar width was observed in patients with vertical pattern.
3. Limited sexual dimorphism was observed in the study population, with females demonstrating smaller condylar length compared to males.

## Data Availability

The datasets generated during and/or analyzed during the current study are available from the corresponding author upon reasonable request.
